# Familial Molecular Burden in Autism Spectrum Disorder: A Next-Generation Sequencing Study of Polish Affected Families

**DOI:** 10.3390/ijms26199672

**Published:** 2025-10-03

**Authors:** Monika Wawszczak-Kasza, Jarosław Rachuna, Łukasz Madej, Wojciech Lewitowicz, Piotr Lewitowicz, Agata Horecka-Lewitowicz

**Affiliations:** 1 Department of Medical Genetics and Laboratory Diagnostics, Collegium Medicum, Jan Kochanowski University in Kielce, 25-369 Kielce, Poland; 2 Collegium Medicum, Jan Kochanowski University in Kielce, 25-369 Kielce, Poland; 3 Faculty of Medical and Health Sciences, Casmir Pulaski Radom University, 26-600 Radom, Poland; 4 Department of Psychiatry, Collegium Medicum, Jan Kochanowski University in Kielce, 25-369 Kielce, Poland

**Keywords:** autism spectrum disorder, ADHD, autism

## Abstract

Autism spectrum disorder (ASD) is a heritable neurodevelopmental condition with a complex genetic architecture. Dissecting the interplay between inherited variants and high-impact de novo variants is critical for understanding its etiology. We conducted a family-based study involving 42 families with ASD (139 individuals). Using a targeted next-generation sequencing (NGS) panel of 236 genes, we identified and characterized rare inherited and de novo variants in affected probands, parents, and unaffected siblings. Our analysis revealed a complex genetic landscape marked by diverse inheritance patterns. De novo variants were predominantly observed in individuals with atypical autism, while biparental (homozygous) inheritance was more common in Asperger syndrome. Maternally inherited variants showed significant enrichment in intronic regions, pointing to a potential regulatory role. We also detected variants in several high-confidence ASD risk genes, including *SHANK3*, *MYT1L*, *MCPH1*, *NIPBL*, and *TSC2*, converging on pathways central to synaptic function and neurogenesis. Across the cohort, five variants of uncertain significance (VUS) were identified, comprising two inherited variants in *ABCC8* and additional variants in *CUL23*, *TSC2*, and *MCPH1*. Our findings underscore the profound genetic heterogeneity of ASD and suggest that distinct genetic mechanisms and inheritance patterns may contribute to different clinical presentations within the spectrum. This highlights the power of family-based genomic analyses in elucidating the complex interplay of inherited and de novo variants that underlies ASD.

## 1. Introduction

Autism spectrum disorder (ASD) is a neurodevelopmental condition characterized by impairments in social communication and the presence of restricted, repetitive behaviors. According to the World Health Organization, approximately 1 in 100 children worldwide is affected by ASD [1]. In the United States, recent surveillance data indicate a prevalence of 1 in 36 among 8-year-old children, with a male-to-female ratio of approximately 4:1 [2]. European estimates vary between 0.6% and 1.5%, reflecting differences in diagnostic practices and healthcare accessibility [3]. While prevalence estimates in Poland remain limited, available data suggest rates comparable to those observed in other European countries [4]. The observed increase in prevalence over recent decades is attributed to changes in diagnostic criteria, improved awareness, and broader access to diagnostic services, rather than a true rise in incidence [5]. The disorder exhibits high heritability, with genetic factors contributing substantially to its etiology [6]. Autism risk is influenced by the cumulative effect of numerous common genetic variants, each contributing a small effect. Genome-wide association studies (GWAS) suggest that these common variants may account for a significant portion of ASD heritability [7]. In some cases, autism may result from the combined effect of a few rare genetic variants with moderate to large effects. This model suggests that multiple rare variants can interact to increase ASD risk [8]. Dedicated databases focusing on the genetic underpinnings of autism, such as SFARI Gene, catalog a wide spectrum of candidate genes. Genes are stratified into three evidence-based tiers: Category 1 (High Confidence), Category 2 (Strong Candidate), and Category 3 (Suggestive Evidence). In addition, SFARI designates a distinct syndromic category, which encompasses genes that confer a substantial increase in risk and are consistently associated with additional clinical features beyond those required for an ASD diagnosis [https://gene.sfari.org, accessed on 12 December 2024].

Careful examination of inheritance is indispensable, since it provides critical insights into the prediction of intricate epigenetic mechanisms underlying ASD. Some families exhibit a pattern where autism is transmitted in a dominant fashion, particularly affecting male offspring. This may occur when a parent, often an unaffected female carrier, passes a variant to her children, with sons being more likely to express the phenotype. [9] In certain populations or consanguineous families, autosomal recessive inheritance has been observed. This involves both parents carrying one copy of a changed nucleotide, with a 25% chance of passing two copies to their child, leading to ASD. Recent studies have significantly expanded our understanding of autosomal recessive inheritance in autism: A 2023 study from University of California, Los Angeles, identified new candidate genes such as *PLEKHA8*, *PRR25*, *FBXL13*, *VPS54*, *SLFN5*, *SNCAIP*, and *TGM1* in families with multiple autistic children [10]. Whole-exome sequencing in Qatari families identified *TRPC4* and *SCFD2* as novel autism-related genes under this inheritance pattern [11,12]. The “Building a Resource for the Advancement of Knowledge of Autism in Qatar” genome project found 13 new candidate genes with autosomal recessive transmission in consanguineous families [13].

Over 150 genes on the X chromosome have now been associated with ASD. Many of these genes are involved in chromatin remodeling, synaptic function, and neuronal signaling, underscoring the genetic complexity of autism and the critical role of the X chromosome [12,14]. Recent studies underscore the contribution of inherited genetic variants transmitted from parents to the overall risk architecture of autism spectrum disorder. Although both rare de novo variants and common polygenic risk factors have been implicated [15,16], the genetic architecture of ASD remains complex and heterogeneous. Recent advances in next-generation sequencing (NGS) have enabled detailed investigation of familial genetic contributions, providing new insights into inherited molecular burdens. In this study, we applied NGS to 42 families with at least one affected individual to explore patterns of inherited and potentially pathogenic variants contributing to ASD susceptibility.

## 2. Results

From 42 families comprising 138 individuals, high-quality genetic material and sequencing data were obtained for 83 participants, including 33 with autism. Only a subset of these variants was classified as rare in the general population and deemed potentially relevant to ASD pathogenesis. Variants annotated as either benign or pathogenic based on ACMG SF v3.1 criteria were highlighted, and their segregation patterns were analyzed within families. We analyzed the localization of each gene on chromosomes to predict the potential mechanism of inheritance, classified as autosomal (46 genes) or X-linked (*HUWE1*).

### 2.1. Candidate Genes in Autism—Insights from DOMINO and SFARI

A total of multiple variants were identified across 47 genes in affected families, with inheritance patterns including maternal, paternal, biparental, and de novo origins. The probability of the inheritance was classified as very likely recessive or likely recessive (30.2%), either dominant or recessive (9.3%), likely dominant or very likely dominant (51.2%) according to DOMINO predictor. Inheritance classification using DOMINO was unavailable for four genes. The selected genes were further classified according to the SFARI framework. Within our cohort, we identified one de novo exonic variant in *HUWE1*. For variants with undetermined parental origin due to insufficient data, we detected one exonic variant in *PCDHA1*, three exonic variants in *PCDHA7*, and one intronic variant in *SNHG14*. The list of genes with DOMINO and SFARI classification was shown in Table 1, Table 2 and Table 3, variants identified exclusively in unaffected siblings were shown in Table 4.

A total of 55 variants were classified by DOMINO analysis as following a dominant mode of inheritance. Among these, eight were maternally inherited, located in *CHD8* (chromatin remodeling, autism), *EN2* (cerebellar development), *SHANK3* (synaptic scaffolding, Phelan–McDermid syndrome), *SCN1A* (sodium channel, epilepsy), *SHANK2* (synaptic scaffolding), *NRXN1* (synapse formation, autism/schizophrenia), and *RAI1* (transcriptional regulation, Smith–Magenis syndrome).

Eight were paternally inherited, including variants in *ARID1A* (chromatin remodeling, neurodevelopment), *CACNA1C* (calcium channel, Timothy syndrome), *EN2*, *SHANK3*, and *RAI1*.

Fifteen were transmitted from both parents, mapping to *TBL1XR1* (transcriptional co-regulator, intellectual disability), *CACNA1C*, *EN2*, *SHANK3*, *SCN1A*, *MYT1L* (neuronal differentiation), *RELN* (neuronal migration, cortical layering), and *NRXN1.*

Of the 22 dominantly inherited genes identified, 18 (81.8%) were classified as Category 1 (High Confidence) in the SFARI Gene database, 2 (9.1%) as Category 2 (Strong Candidate), and 1 (4.5%) as Category 3 (Suggestive Evidence). In addition, 12 genes (54.5%) were associated with genetic syndromes and therefore categorized as syndromic (S).

Among variants with unclear inheritance, we detected only one intronic variant in *CACNA2D3* of maternal origin. *CACNA2D3* encodes the α2δ3 subunit of voltage-dependent calcium channels, which plays a critical role in regulating calcium influx into neurons and modulating synaptic transmission. It should be noted that three out of four genes were classified as Category 1 (High Confidence) according to the SFARI Gene database, and in two cases, an association with genetic syndromes was identified.

In the case of variants inherited in a recessive manner, 14 were of maternal origin, located in *ABCC8* (insulin regulation), *TPH2* (serotonin synthesis), *NSUN2* (RNA modification), *SYNE1* (ataxia), *STIL* (cell division/brain development), *SCN7A* (sodium channel), *TRAPPC9* (intellectual disability), and *VPS13B* (Cohen syndrome). Six were of paternal origin, identified in *ABCC8*, *NSUN2*, *WFS1* (Wolfram syndrome), and *STIL*. Additionally, 12 were transmitted from both parents, including seven cases in *TPH2*, as well as variants in *MCPH1* (DNA repair/brain size), *STIL*, and *VPS13B*. In this case, the vast majority of genes had no established association with ASD according to the SFARI Gene database. Among the 13 identified genes, 2 (15.4%) were classified as Category 1 (High Confidence), 3 (23.1%) as Category 2 (Strong Candidate), while 3 (23.1%) were linked to genetic syndromes.

The variants identified exclusively in the healthy siblings were intronic, three of which followed a recessive mode of inheritance. These occurred in *MAGEL2* (neurodevelopment), *CACNA1H* (neuronal excitability), *SLC9A9* (synaptic regulation), and *ASPM* (cortical development). Their intronic location and inheritance pattern indicate limited clinical relevance. According to the SFARI Gene classification, one gene was assigned to Category 1 (High Confidence) and was also linked to a genetic syndrome, while two genes were classified as Category 2 (Strong Candidate)

When considering all variants collectively, we observed a heterogeneous pattern of inheritance. In total, 23 maternally and 14 paternally inherited variants were identified, spanning both exonic and intronic regions as well as one located in the untranslated region (UTR). In two cases, the parental origin remained ambiguous. Furthermore, 12 de novo variants were detected, distributed across exonic and intronic regions. Of note, four intronic variants were found exclusively in healthy siblings, suggesting limited clinical relevance given their genomic context and inheritance pattern.

### 2.2. Variant of Uncertain Significance

Pathogenicity assessment using the Franklin database (https://franklin.genoox.com/, accessed on 20 May 2025) classified five variants as VUS (Variant of Uncertain Significance). The results are summarized in Table 5.

Two variants were identified in the *ABCC8* gene: p.Ala726Thr, inherited from the father, and p.Ser1053Asn, inherited from the mother. Both fulfilled the PM2 criterion, defined as absence or extremely low frequency in large population databases (e.g., gnomAD), and were categorized as PP2, consistent with the low tolerance of *ABCC8* to missense variation. In addition to these, a variant in the *CUL23* gene (p.Gln343Arg) was identified. This variant also fulfilled PM2, was classified as PP3 by in silico predictions supporting a deleterious effect, and PP2 based on intolerance of the gene to missense changes. The same variant was also present in a healthy sibling. In the *TSC2* gene, the variant p.Arg1529Gln was identified in one affected proband as well as in a healthy sibling. This variant was classified as PM2 and PP3, reflecting both rarity in population databases and in silico predictions supportive of a deleterious effect.

A synonymous variant in *MCPH1* (p.Arg171=) was identified in three affected children in the homozygous state. This variant was also classified as PM2 based on its absence or extremely low frequency in population databases.

### 2.3. Segregation Analysis

**Figure 1:**
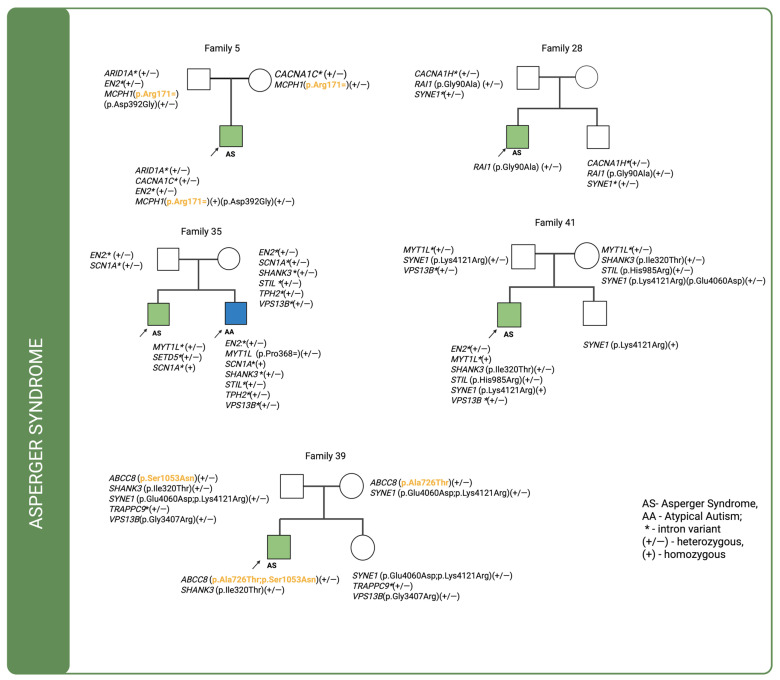
Pedigrees of individuals diagnosed with Asperger syndrome (ICD-10: F84.5) for whom sequencing data were available for both the proband and their parents, illustrating the inheritance patterns of identified genetic variants. VUS—highlighted in orange and bolded.

**Figure 2:**
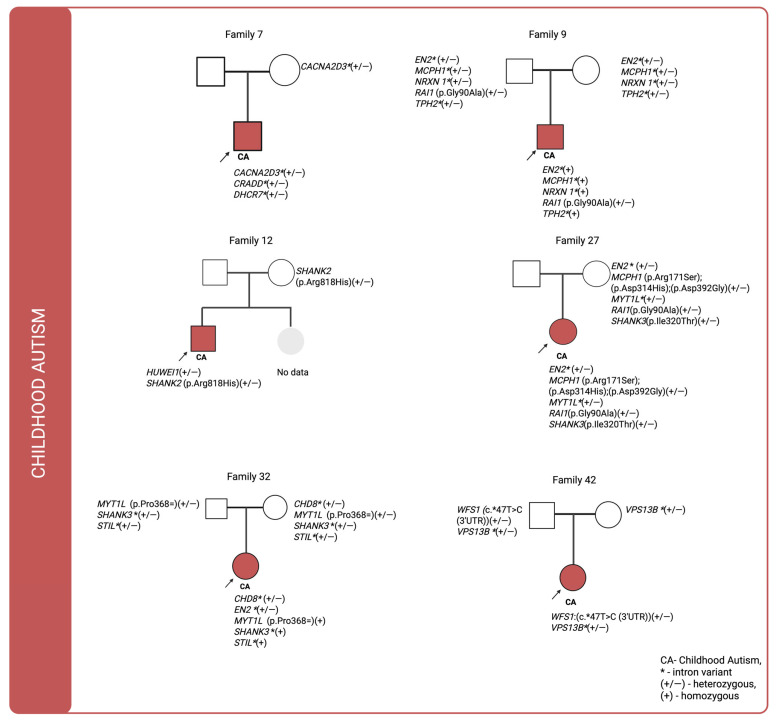
Pedigrees of individuals diagnosed with childhood autism (ICD-10: F84.0) for whom sequencing data were available for both the proband and their parents, illustrating the inheritance patterns of identified genetic variants.

**Figure 3:**
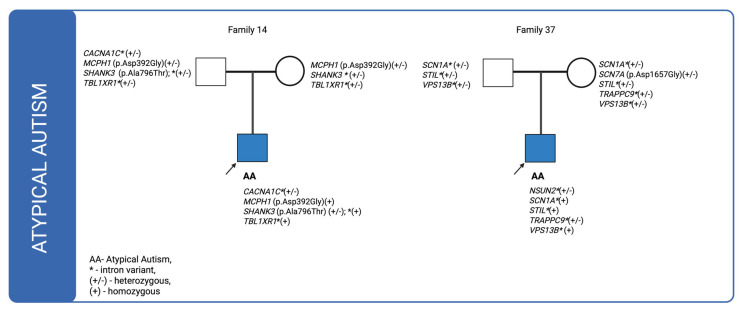
Pedigrees of individuals diagnosed with atypical autism (ICD-10: F84.1) for whom sequencing data were available for both the proband and their parents, illustrating the inheritance patterns of identified genetic variants.

For cases in which multiple genetic variants were detected and sequencing data were available for both affected individuals and their parents, we investigated the co-segregation of these variants in the context of their inheritance modes. The corresponding pedigrees are shown in Figure 1, Figure 2 and Figure 3. In the figures, variants classified as VUS in the Franklin database are highlighted in orange and bolded, whereas the remaining variants are shown as likely benign.

In four out of five Asperger syndrome families, variants were inherited from both parents, with intronic variants being the most prevalent. In family 5, a missense variant in *MCPH1* (p.Asp392Gly) was inherited exclusively from the father, together with a synonymous variant (p.Arg171=). The synonymous variant is classified as a VUS (Variant of Uncertain Significance). DOMINO analysis predicts a recessive mode of inheritance, consistent with the segregation pattern in this family, where two altered alleles were transmitted by healthy parents to the affected child. Furthermore, the SFARI Gene database assigns *MCPH1* to category 2, indicating strong evidence for its implication in autism spectrum disorder from multiple independent genetic studies, although additional replication and functional validation are warranted to confirm its pathogenic role. In family 28, no maternally inherited variants were identified in the analyzed genes; a missense variant in the *RAI1* gene was inherited from the father but was also present in the proband’s unaffected brother. In family 35, which included two affected boys—one diagnosed with Asperger syndrome and the other with atypical autism—only intronic variants were found to be inherited. In the child with Asperger syndrome, no missense variant were identified in the analyzed genes. In contrast, the child with atypical autism carried three de novo variants: a synonymous variant in the *MYT1L* (p.Pro368=) gene. In family 39, two missense variants in *ABCC8* (p.Ala726Thr and p.Ser1053Asn) were identified, each inherited from a different parent, resulting in a compound heterozygous genotype in the proband. Both variants are classified as VUS, yet their combined effect may contribute to the disease phenotype. Consistent with this observation, DOMINO analysis predicts a recessive mode of inheritance for *ABCC8*, supporting the interpretation that the presence of two deleterious alleles, transmitted independently from healthy parents, may underlie the proband’s condition. Notably, *ABCC8* is not listed among autism risk genes in the SFARI Gene database, underscoring the novelty of this finding in the context of neurodevelopmental disorders. Additionally, a paternally inherited variant was detected in the *SHANK3* (p.Ile320Thr) gene. None of these variants was present in the proband’s unaffected sister. In family 41, two missense variants—*STIL* (p.His985Arg) and *SHANK3* (p.Ile320Thr)—were inherited from the mother and were absent in the unaffected brother. A *SYNE1* (p.Lys4121Arg), variant inherited from the father, was present in both siblings.

Pedigree analysis of individuals diagnosed with childhood autism (ICD-10: F84.0) revealed that in three of six cases, no paternally inherited variants were detected, whereas maternally inherited variants were present in all of them. These variants were predominantly intronic. De novo variants identified in families 7, 12, and 32 were primarily located within intronic loci. In family 9, a missense variant in the *RAI1* (p.Gly90Ala) gene was inherited from the father, while only intronic variants were transmitted from the mother. In family 12, no variants were identified in the father’s genome that were passed on to the offspring. A variant potentially affecting amino acid synthesis in *SHANK2* (p.Arg818His) was inherited from the mother. Notably, in family 27 there were three maternally inherited missense variants in *MCPH1*(p.Arg171Ser;p.Asp314His;p.Asp392Gly). In family 42 a variant located in the untranslated region (UTR) of the WFS1 gene and inherited from the father was identified.

In family 14, a missense variant in the *SHANK3* (p.Ala796Thr) gene was inherited from the father, while a variant in *MCPH1* (p.Asp392Gly) was transmitted from both parents, resulting in a homozygous genotype in the affected individual. Case 35, illustrated in Figure 1, also involves a patient with atypical autism who has an affected sibling. The patient inherited intronic variants from the father and three de novo variants were identified: one synonymous variant in *MYT1*(p.Pro368=) and two missense variants in *NIPBL* (p.Ala179Thr; p.Asn968Ser). In the case of family 37 only intronic variants were found to be inherited. Notably, one of these, located in *NSUN2*, was classified as de novo.

### 2.4. Variant Segregation Among Siblings

In our cohort, we identified a heterogeneous spectrum of variants across different family structures; the data are shown in Table 6. The table presents results from families in which sequencing was of high quality and available for both the affected proband and their siblings.

Among nine two-child families with one affected and one unaffected sibling, most variants were observed exclusively in the affected child, including *ABCC8* (p.Ser1053Asn), *SHANK3* (p.Ile320Thr), and *STIL* (p.His985Arg). In several families, variants were shared between siblings, either in both (*RAI1* p.Gly90Ala) or restricted to the unaffected child (*ANKRD11* p.Arg840Gln, *SLC9A9* p.Asn43Lys, *SYNE1* p.Ser4596Thr, p.Lys4121Arg). In two three-child families with a single affected proband, variants showed partial segregation, with examples present in both the proband and one unaffected sibling (*CUL3* p.Gln343Arg, *POGZ* p.His1363Gln, *NRXN1* c.*98A>G), while others were confined to the proband (*ANK2*, *HDAC4*, *KCNJ11*). Finally, in one two-child family with both children affected, we identified a shared *MYT1L* synonymous variant (c.1104C>A, p.Pro368=). Notably, several of the detected variants represent VUS, including *ABCC8* (p.Ala726Thr), *CUL3* (p.Gln343Arg), *VPS13B* (p.Val3780Leu), and *TSC2* (p.Arg1529Gln). These VUS were identified both in affected and, in some cases, unaffected siblings, reflecting incomplete segregation within families. The presence of VUS in genes implicated in neurodevelopment highlights potential relevance but precludes definitive interpretation without additional functional or segregation data.

### 2.5. Family History of Psychiatric Disorders

**Figure 4:**
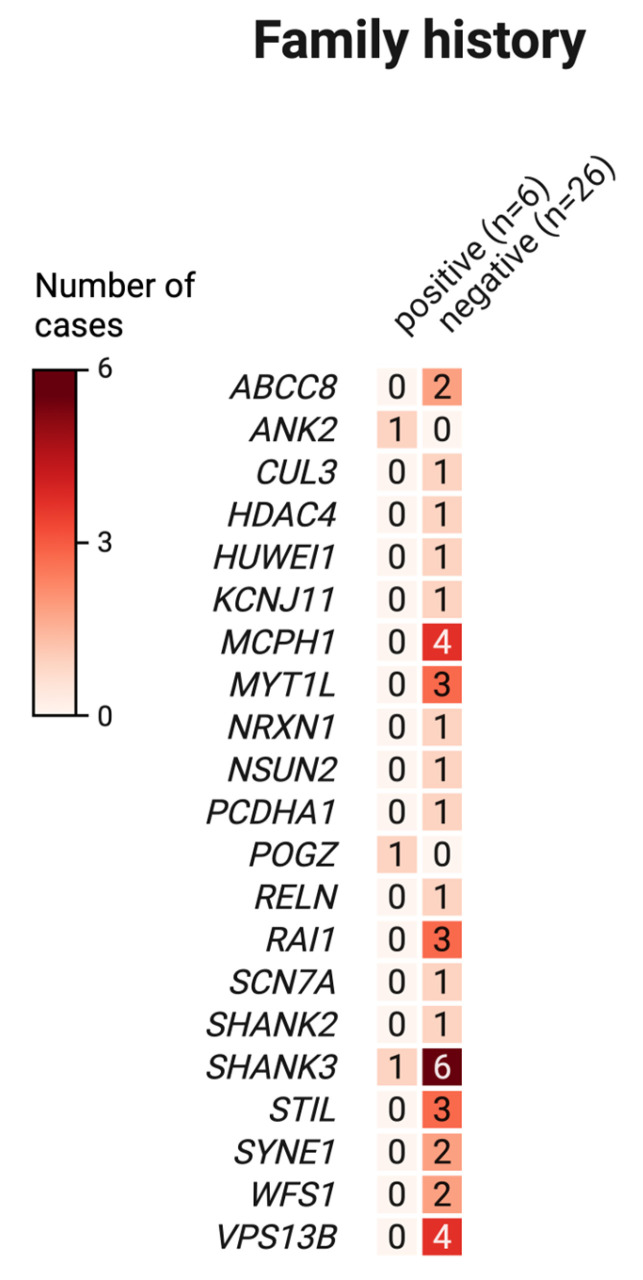
Distribution of exonic genetic variants in families with and without a positive family history of psychiatric disorders.

We additionally examined the prevalence of psychiatric disorders among family members, focusing on schizophrenia (ICD-10 code F20) and bipolar affective disorder (ICD-10 code F31). Evidence of these conditions was identified in 16,7% (7 of 42) of families assessed. To explore potential genetic correlations, we compared the distribution of genetic variants between individuals with and without a documented family history of these psychiatric disorders (de novo variants were excluded from the analysis). The results are shown in Figure 4. Sequencing data were available for six families with a positive family history and for twenty-six families without such a history.

We found no evidence of an association between family history and the distribution of genetic variants in the analyzed groups.

## 3. Discussion

Despite decades of research, the genetic basis of autism remains largely unresolved. De novo variants and large CNVs explain only a small subset of cases, while inherited variants of modest effect may interact with these events to influence risk. Such de novo variants, often highly penetrant in males, are thought to drive a considerable fraction of sporadic autism [9,17,18]. This pattern further supports the notion of a sex-specific liability threshold in ASD, whereby females may require a greater cumulative genetic burden to manifest the disorder. In our cohort of 43 ASD cases, 39 were male (90.7%) and 4 were female (9.3%), a distribution consistent with the pronounced male predominance reported across numerous studies. Inherited variation, while generally of lower penetrance, can interact with spontaneous changes to influence disease expression, especially in simplex families. By our family-based genomic investigation into the genetic underpinnings of ASD, we successfully identified a diverse landscape of variants across 47 candidate genes, allowing for a detailed examination of their inheritance patterns. Among these, we identified five variants classified as VUS (Variants of Uncertain Significance), distributed across four genes, as well as additional variants classified as likely benign and observed at varying frequencies in the population. Together, these findings highlight both the complexity of variant interpretation and the challenges in delineating their contribution to disease risk.

While the use of non-invasive buccal swabs presented challenges in DNA quality that precluded the definitive assignment of inheritance for every detected variant, our analysis of high-confidence calls revealed distinct and informative patterns of genetic transmission. A significant number of variants were traced to a maternal origin, affecting genes implicated in biological processes fundamental to neurodevelopment. Notably, we identified maternally inherited missense variants in genes crucial for synaptic signaling and structure, such as *SHANK2*, *SHANK3*, and *NRXN1*, which are well-established risk factors for ASD [19,20]. Further variants were identified in critical neurodevelopmental regulators, including *CHD8* and *RAI1*, as well as in genes involved in ion channel function (*ABCC8*, *SCN7A*) and key metabolic or structural pathways (*VPS13B*, *STIL*, *SYNE1*). The DOMINO classification predicted a dominant mode of inheritance for the core synaptic genes *SHANK2*, *SHANK3*, and *NRXN1*, indicating that even a single altered allele could contribute to ASD liability. It is worth noting that 9 out of 17 maternally inherited genes (52.9%) were classified as Category 1 (High Confidence) in the SFARI Gene database, while 3 (17.6%) were assigned to Category 2 (Strong Candidate). Four genes (23.5%) were associated with genetic syndromes. Although SFARI classification applies to genes rather than individual variants, it provides a useful framework for selecting molecular targets for panel-based studies. In our cohort, the respective variants were classified as likely benign; however, this does not exclude their potential contribution to disease through interaction with other genetic alterations or environmental factors.

Paternally inherited variants overlapped with maternally implicated genes, with missense changes observed in *SHANK3*, *RAI1*, and *ABCC8*, suggesting that these loci may represent points of vulnerability independent of parental origin. Consistent with this, heterozygous *SHANK2* variants disrupt synapse formation and neuronal morphology, contributing to autistic traits [20], while SHANK3 and NRXN1 variants have also been reported in ASD-affected children [19]. Similarly, *CHD8* has emerged as a central regulator of neurogenesis, with de novo variants strongly associated with autism [21].

The functional role of *ABCC8*, which encodes the sulfonylurea receptor 1 (SUR1)—a regulatory subunit of ATP-sensitive potassium (_K_ATP) channels—further underscores the potential importance of ion channel genes. By coupling cellular metabolism to membrane excitability in both pancreatic β-cells and neurons, disruptions of _K_ATP channel function may alter excitability and signaling, a mechanism that could be relevant in neurodevelopmental disorders such as ASD [22,23].

Variants were also detected in *WFS1*, including one in the 3′ untranslated regions (3′UTR), potentially affecting post-transcriptional regulation. While no conclusive evidence links *WFS1* directly to ASD, this gene is well established as the cause of Wolfram syndrome—a recessive neurodegenerative disorder characterized by diabetes mellitus, optic atrophy, and hearing loss—as well as autosomal dominant forms of non-syndromic deafness [24]. Although isolated reports have described autism-like traits in individuals with *WFS1* variants, the evidence remains limited and inconclusive [25]. According to DOMINO, *WFS1* variants are typically predicted to follow a recessive inheritance pattern; however, clinical observations, particularly in DFNA6 (a non-syndromic form of low-frequency sensorineural hearing loss), demonstrate that certain *WFS1* alleles can act dominantly. This duality in inheritance underscores the allele-specific nature of *WFS1* pathogenicity, likely shaped by dosage sensitivity and tissue-specific expression, and raises the possibility that dominant-acting variants could, under certain conditions, contribute to neuropsychiatric phenotypes. Perhaps one of the most compelling findings of our study was the identification of homozygous missense variants, which points to a biparental inheritance pattern, in four critical neurodevelopmental genes: *SHANK3*, *MYT1L*, *MCPH1*, and *VPS13B*. A particularly heavy burden was observed in *MCPH1*, a gene encoding a centrosomal protein vital for neurogenesis, where we documented seven distinct missense changes. The classification of *MCPH1* as a strong candidate gene for ASD by the SFARI database, combined with the recessive inheritance model predicted by DOMINO, strongly aligns with our findings and supports the pathogenic potential of these homozygous variants. In our study, we identified a synonymous variant in *MCPH1*(p.Arg171=), classified as a VUS, which was present in three affected children in the homozygous state, further underscoring its potential relevance in disease etiology. We also identified, homozygous variants in *MYT1L*, a high-confidence ASD risk gene that encodes a transcription factor essential for establishing and maintaining neuronal identity, further underscore the theme of disrupted core neurodevelopmental processes. Beyond these clearer patterns, our analysis also uncovered more complex instances of inheritance that highlight the multifaceted nature of genetic risk. For example, in one proband from Family 39, we identified two different missense variants within the *ABCC8* gene (p.Ala726Thr; p.Ser1053Asn). One was inherited from the mother and the other from the father, representing a classic biallelic inheritance pattern often associated with recessive conditions or synergistic effects. Importantly, these variants were not identified in the unaffected sibling, further supporting their potential relevance to disease manifestation. Both variants were classified as VUS and occur at very low frequencies in population databases, consistent with potential pathogenic relevance. The co-occurrence of two rare missense variants in trans is particularly noteworthy, as it highlights a possible cumulative effect on protein function and underscores the importance of considering compound heterozygosity in the genetic architecture of ASD Collectively, these findings paint a detailed picture of a complex genetic architecture in ASD, where maternal, paternal, and biparental inheritance patterns all contribute to the landscape of risk, acting through genes critical to synaptic function and brain development. The potential involvement of some genes, such as *WFS1*, *ABCC8*, and *SCN7A*, remains speculative and should be regarded as hypotheses requiring further validation rather than definitive associations.

In addition, we identified homozygous variants in *MYT1L*, which encodes a neuron-specific transcription factor essential for maintaining neuronal identity and regulating neurodevelopmental gene expression. Loss-of-function variants in *MYT1L* have been recurrently described in individuals with neurodevelopmental disorders, including ASD, intellectual disability, and obesity. Supporting its causal role, Weigl et al. (2023) demonstrated that *MYT1L* haploinsufficiency in mice leads to disrupted cortical development, altered synaptic gene expression, and ASD-like phenotypes such as impaired social interactions and increased neuronal excitability [26]. Collectively, these findings underscore *MYT1L* as a high-confidence neurodevelopmental risk gene with direct relevance to ASD.

### 3.1. De Novo Variants

Multiple studies have demonstrated that de novo variants contribute substantially to ASD risk, as evidenced by comparisons between probands and unaffected siblings showing an excess burden of protein-altering de novo variants in affected individuals. These variants often occur in genes with established roles in neurodevelopment, synaptic architecture, and neuronal signaling. For example, O’Roak et al. (2014) identified recurrent de novo variants in *CHD8*, *DYRK1A*, and *SCN2A* in simplex families, where only one child is affected by ASD [18]. Such findings support a model in which highly penetrant de novo events in genes critical to brain development act as primary drivers of sporadic cases.

In our cohort, several compelling de novo missense variants were identified in probands within genes strongly implicated in neurodevelopmental disorders, including *NIPBL*, *TSC2*, *MYT1L*, *CRADD*, and *HUWE1*. The clinical relevance of these findings is underscored by the established roles of these genes in related syndromes: *NIPBL* is the major causal gene in Cornelia de Lange syndrome, frequently associated with autistic features; pathogenic variants in *TSC2* cause Tuberous Sclerosis Complex, which is highly comorbid with ASD [27,28,29,30,31,32]; and *HUWE1* is an X-linked gene associated with intellectual disability and ASD-like phenotypes [33]. Notably, within TSC2, we identified a variant classified as VUS (p.Arg1529Gln), which, despite its current uncertain significance, is of particular interest given the critical role of TSC2 in mTOR pathway regulation and neurodevelopment. The presence of de novo variants in these functionally essential genes strongly supports their potential pathogenic contribution to ASD in our cohort.

Beyond cohort-level observations, our study also enabled a detailed intra-familial comparison in a multiplex family with two siblings diagnosed on the autism spectrum. Here, we observed both shared inherited and unique de novo variants contributing to divergent phenotypes. The first sibling, diagnosed with Asperger syndrome, carried two de novo intronic variants in *MYT1L* and *SETD5*, together with a homozygous intronic variant in *SCN1A* inherited from both parents. The second sibling, diagnosed with atypical autism, presented four distinct de novo variants—an intronic change in *EN2*, a synonymous variant in *MYT1L*, in addition to the same homozygous *SCN1A* variant shared with the brother. Although these variants were individually classified as benign or likely benign, this does not diminish their potential importance, as ASD is increasingly regarded as a multifactorial condition in which such variants may exert a cumulative effect or interact with additional genetic and environmental factors. These findings highlight the coexistence of common inherited risk factors and distinct de novo events within the same family, underscoring the complex interplay between background genetic susceptibility and individual mutational landscapes in shaping ASD phenotypes.

### 3.2. Family History of Mental Disorders

The investigation of family history in relation to ASD risk supports a significant association between these factors. Lin et al. reported that the prevalence of schizophrenia or bipolar disorder was 1.8% among typically developing children, compared with 5.2% in those diagnosed with ASD [30]. In our cohort, the proportion was even higher, with 16.67% of children having a positive family history.

Consistent with these observations, Lin and colleagues further noted that a family history of schizophrenia is associated with nearly a threefold increase in the risk of ASD, while bipolar disorder confers a more moderate, though still elevated, risk. Findings from the large cross-disorder genome-wide meta-analysis conducted by the Cross-Disorder Group of the Psychiatric Genomics Consortium provide compelling evidence that genetic risk factors for major psychiatric conditions transcend traditional diagnostic categories. In that study, nearly 75% of genome-wide significant loci exhibited pleiotropic effects, with the strongest genetic correlation (rg, i.e., the genome-wide genetic correlation coefficient) observed between schizophrenia (SCZ) and bipolar disorder (BD), estimated at approximately 0.70. Autism spectrum disorder (ASD) also demonstrated significant, albeit more moderate, overlap with both SCZ and BD. Recurrent copy-number variants, including [30,31] those at 15q11.2 and 17q12, were implicated across ASD and SCZ, highlighting convergent molecular pathways involved in neurodevelopment and synaptic regulation [31].

Evidence from large population-based studies underscores the role of shared genetic susceptibility across these disorders [30,31].

### 3.3. Study Limitations

This study has several limitations. First, the biological material obtained from affected children (buccal swabs) was frequently of suboptimal quality and often yielded insufficient cellular content for high-confidence analysis. Second, the available family history data on psychiatric disorders lacked clinical specificity and detail, limiting their utility for stratified genetic comparisons. Third, although whole-genome sequencing (WGS) provides a comprehensive assessment of genetic variation, its full potential cannot be realized due to sample quality constraints. Fourth, the sample size was modest, limiting statistical power and generalizability, and should be expanded in future work. Finally, while DOMINO offers valuable predictions of inheritance patterns, its accuracy depends on previously annotated genes and may be less reliable for poorly characterized loci. These constraints highlight the importance of cautious interpretation and underscore the need for independent experimental validation.

In summary, our study highlights the significant contribution of both de novo and inherited genetic variation to autism spectrum disorder (ASD), with particular emphasis on genes implicated in synaptic function and early neurodevelopment. Variants exhibited diverse inheritance patterns—maternally, paternally, biparentally, or arising de novo. Maternally inherited variants were more frequently located within intronic regions, whereas de novo variants occurred across both coding and non-coding sequences. Paternally inherited variants included both exonic and regulatory changes, with several affecting genes previously associated with neurodevelopmental phenotypes, suggesting a potential pathogenic role. Biparentally inherited variants, often identified in a homozygous state, were enriched in genes predicted to follow a recessive mode of inheritance, supporting their possible involvement through compound heterozygosity or dosage effects.

## 4. Materials and Methods

Design: A total of 42 ASD-affected families were included in the study. Among the 43 affected children, 39 were boys (90.7%) and 4 were girls (9.3%). Family structures were distributed as follows: among the 43 families included in the study, 31 (72.1%) had a single affected child with ASD, 9 (20.9%) comprised one affected child and one unaffected sibling, 2 (4.7%) had three children of whom only one was affected, and 1 family (2.3%) had two children diagnosed with ASD. In two cases the DNA concentration was insufficient, and these samples were excluded from analysis. In one family the biological father was absent, and in another one father refused a contribution in research. Finally, we collected 139 samples. The diagnosis of ASD was made by a specialist of child and adolescent psychiatrist according to DSM-5 rules.

Sample collection and DNA isolation: The genomic DNA was collected from volunteers according to buccal swab collection protocol. The patient rinses the mouth with water for at least 30 s, to remove any food particles or contaminates that might affect sample quality. At least 1 min was required to collect desquamated epithelial cells from the oral mucosa. The collected swab was put into the probe with a lysis buffer immediately. The isolation was conducted using gravity-flow method (Genomic Micro AX Swab Gravity Plus, A&ABiotechnology, Gdansk, Poland) according to manufacturers. DNA concentration was determined using spectrophotometric method (DeNovix DS-11+, DeNovix, Wilmington, DE, USA) and fluorometric method (QuantiFluo^®^ ONE dsDNA System (Promega, Madison, WI, USA). Good quality DNA (min. 10 ng/µL) was further analyzed. The identification of genetic variants in 236 genes associated with autism was conducted using NGS method (Illumina Autism Research Panel) The full list of analyzed amplicons is available on manufacturer’s website (https://emea.illumina.com/products/by-brand/ampliseq/community-panels/autism.html, accessed on 1 September 2022).

Library preparation and Sequencing Libraries were constructed using the AmpliSeq for Illumina Library Prep (Illumina, San Diego, CA, USA) according to the manufacturer’s instructions. Validation and Quantification of the libraries were performed using quantitative PCR (qPCR) protocol. Library normalization and pooling were prepared according to AmpliSeq for Illumina Autism Research Panel. Sequencing was carried out using the MiSeq sequencing platform (Illumina, Inc., San Diego, CA, USA). Using MiSeq Reagent Kits v2, (Illumina, Inc., San Diego, CA, USA).

Data analysis: The quality analysis of raw FASTQ data was performed using FastQC version 0.12.0 [https://www.bioinformatics.babraham.ac.uk/projects/fastqc/] (accessed on 12 December 2024). Based on the analysis, data quality was assessed, and criteria were established for discarding low-quality reads. Additionally, a decision was made to trim the first 17 nucleotides due to poor sequencing quality using the fastx-Toolkit software [https://github.com/agordon/fastx_toolkit] (accessed on 12 December 2024). The report did not indicate any overrepresented sequences. Read mapping was carried out using Bowtie2 version 2.5.4 [32] to the hg19 reference genome provided by the UCSC Genome Browser [hgdownload.soe.ucsc.edu/goldenPath/hg19/bigZips] (accessed on 12 December 2024). Duplicate marking and removal were then performed using Picard:MarkDuplicates [broadinstitute.github.io/picard] (accessed on 12 December 2024).

SNP and INDEL Variant Calling and Annotation: To call germline variants, a pileup file was first generated using Samtools: samtools mpileup -B -f [reference sequence] [BAM file] >myData.pileup [34]. Genetic variants were called using VarScan 2 [https://varscan.sourceforge.net/, accessed on 12 December 2024] with the mpileup2 protocol for variants with at least x8 coverage --min-coverage 8, minimum frequency 0.01 --min-var-freq 0.01, and statistical significance greater than 0.05 --*p*-value 0.05. The filtering criteria were the same for single nucleotide variants and structural variants. The detected variants were annotated using the web-based version of wAnnovar in relation to the hg19 reference genome [35]. Annotated variants were cross-referenced with established databases including VarSome, ClinVar, gnomAD, dbSNP, SnpEff, and LiftVar. Variant classification was assigned according to the American College of Medical Genetics and Genomics Secondary Findings Version 3.1 (ACMG SF v3.1) guidelines. All identified variants were additionally verified using the Franklin platform (Genoox; https://franklin.genoox.com/) (accessed on 20 May 2025).

Statistical analysis and data visualization: Statistical analysis was conducted using Statistica software [Statsoft Poland, v.13.3.7] and R [v 4.1.2, R Core Team (2021). R: A language and environment for statistical computing. R Foundation for Statistical Computing, Vienna, Austria. URL https://www.R-project.org/, accessed on 12 December 2024]. The probability of inheritance was estimated using DOMINO, a tool assessing the likelihood for a gene to harbor dominant changes. The method is based on linear discriminant analysis (LDA) trained on a set of genes with known inheritance mode on series of specific features, and finally validated with an independent group of genes [36]. The figures were created using BioRender (https://biorender.com) (accessed on 30 May 2025).

## 5. Conclusions

Importantly, distinct inheritance patterns were observed across ASD subtypes. Biparental inheritance was predominant among individuals with Asperger syndrome, whereas de novo missense and synonymous variants were more frequently detected in cases of atypical autism. In childhood autism, maternally inherited variants were consistently observed, while paternally inherited variants were absent in multiple cases. Inherited variants shared with unaffected siblings point to incomplete penetrance, whereas de novo variants were exclusive to affected individuals, supporting their potential causal role. These findings reinforce the genetic heterogeneity underlying ASD and underscore the importance of continued investigation in larger, well-characterized cohorts. Integration of whole-genome sequencing (WGS) will be critical to capture non-coding and regulatory variation and to advance our understanding of the molecular mechanisms contributing to ASD pathogenesis.

## Figures and Tables

**Table 1:** Genes with Reported Variants: Location, Probable Inheritance Type Classified as Very likely dominant or likely dominant in DOMINO Score range from 0.60 to 0.99, and Number of Affected Individuals. I—intron, E—exon, UTR—untranslated region. S—syndromic, 1—High Confidence, 2—Strong Candidate, 3—Suggestive Evidence, ND—no data.

Gene ID	SFARI	Chromosomal Location	Maternal	Paternal	Biparental	De novo	Not Determined
*ARID1A*	3/S	1p36.11	0	1(I)	0	1(I)	0
*TBL1XR1*	1	3q26.32	0	0	1(I)	0	0
*TSC1*	1/S	9q34.13	0	0	0	0	1(E)
*CHD8*	1/S	14q11.2	1(E)	0	0	0	0
*HDAC4*	2/S	2q37.3	0	0	0	0	1(E)
*NIPBL*	1/S	5p13.2	0	0	0	1(E)	0
*CACNA1C*	1/S	12p13.33	0	1(I)	2(I)	0	0
*CUL3*	1	2q36.2	0	0	0	0	1(E)
*EN2*	2	7q36.3	1(I)	1(I)	3(I)	2(I)	1(I)
*SHANK3*	1/S	22q13.33	2(E/I)	3(E)	3(2I/1E)	0	1(I)
*SCN1A*	1/S	2q24.3	1(I)	0	2(I)	0	1(I)
*TSC2*	1/S	16p13.3	0	0	0	1(E)	1(E)
*ANK2*	1	4q25–4q26	0	0	0	0	2(E)
*SHANK2*	1	11q13.3–11q13.4	1(E)	0	0	0	0
*MYT1L*	1	2p25.3	Maternal or paternal 1(I)		2(E)	2(E/I)	1(I)
*ANK3*	1	10q21.2	0	0	0	0	1(E)
*RELN*	1	7q22.1	0	0	1(I)	0	1(E)
*KCNJ11*	ND	1p15.1	0	0	0	0	1(E)
*POGZ*	1/S	1q21.3	0	0	0	0	1(E)
*KMT2C*	1/S	7q36.1	0	0	0	0	1(E)
*NRXN1*	1	2p16.3	1(E)	0	1(I)	0	1(UTR)
*RAI1*	1/S	17p11.2	1(E)	2(E)	0	0	0

**Table 2:** Genes with Reported Variants: Location, Probable Inheritance Type Classified as either dominant or recessive in DOMINO Score range from 0.40 to 0.59, and Number of Affected Individuals. I—intron, E—exon. S—syndromic, 1—High Confidence, ND—no data.

Gene ID	SFARI	Chromosomal Location	Maternal	Paternal	Biparental	De novo	Not Determined
*CACNA2D3*	1	3p21.1–3p14.3	1(I)	0	0	0	0
*ANKRD11*	1/S	16q24.3	0	0	0	0	1(E)
*SETD5*	1/S	3p25.3	0	0	0	1(I)	0
*KCNJ10*	ND	1q23.2	0	0	0	0	1(E)

**Table 3:** Genes with Reported Variants: Location, Probable Inheritance Type Classified as likely recessive or very likely recessive in DOMINO Score range from 0.0 to 0.39, and Number of Affected Individuals. I—intron, E—exon, UTR—untranslated region. S—syndromic, 1—High Confidence, 2—Strong Candidate, ND—no data.

Gene ID	SFARI	Chromosomal Location	Maternal	Paternal	Biparental	De novo	Not Determined
*ABCC8*	ND	11p15.1	1(E)	1(E)	0	0	1(E)
*TPH2*	ND	12q21.1	2(I)	0	1(I)	0	0
*NSUN2*	ND	5p15.31	1(I)	1(I)	0	1(I)	0
*CNTNAP2*	2/S	7q35–7q36.1	0	0	0	0	1(I)
*CRADD*	ND	12q22	0	0	0	1(E)	0
*SYNE1*	2	6q25.2	3(2E/1I)	0	0	0	0
			Maternal or paternal (1E)				
*MCPH1*	2	8p23.1	0	0	7(E)	0	0
*WFS1*	ND	4p16.1	0	3(I/E/UTR)	0	0	0
*DHCR7*	1/S	11q13.4	0	0	0	1(I)	0
*STIL*	ND	1p33	3(E)	1(I)	2(I)	0	0
*SCN7A*	ND	2q24.3	1(E)	0	0	0	0
*TRAPPC9*	2	8q24.3	1(I)	0	0	0	0
*VPS13B*	1/S	8q22.2	2(E)	0	2(E)	0	1(E)

**Table 4:** Genes with Reported Variants: Location, Number of cases healthy siblings. I—intron. S—syndromic, 1—High Confidence, 2—Strong Candidate, ND—no data.

Gene ID	Chromosomal Location	SFARI	DOMINO Score	DOMINO Classification	Maternal	Paternal	Biparental	De novo	Not Determined
*MAGEL2*	15q11.2	1/S	0.8018	very likely dominant	0	0	0	0	1(I)
*CACNA1H*	16p13.3	2	0.3915	likely recessive	0	1(I)	0	0	0
*SLC9A9*	3q24	2	0.07653	very likely recessive	0	0	0	1(I)	0
*ASPM*	1q31.3	ND	0.06048	very likely recessive	0	0	1(I)	0	0

**Table 5:** Classification of variants of uncertain significance (VUS) identified in the cohort. - no data.

Gene	Nucleotide Change (HGVS)	Protein Change (HGVS)	Population Data	In Silico Prediction	Functional Data	
*ABCC8*	NM_000352.6:c.2176G>A	p.Ala726Thr	PM2	-	PP2	
*ABCC8*	NM_000352.6:c.3158G>A	p.Ser1053Asn	PM2	-	PP2	
*CUL3*	NM_003590.5:c.1028A>G	p.Gln343Arg	PM2	PP3	PP2	
*TSC2*	NM_000548.5:c.4586G>A	p.Arg1529Gln	PM2	PP3	-	In healthy siblings
*MCPH1*	NM_024596.5:c.513G>A	p.Arg171=	PM2	-	-	
*VPS13B*	NM_152564.5 c.11338G>T	p.Val3780Leu	PM2	-	-	In healthy siblings

PM2 (Population data): variant absent or extremely rare in population databases (gnomAD), according to Franklin classification, suggesting potential pathogenic relevance. PP3 (In silico prediction): multiple computational algorithms (as implemented in Franklin) predict a deleterious effect on the gene or protein. PP2 (Functional data): variant occurs in a gene with low tolerance for missense variation, where pathogenic missense variants are a common disease mechanism, as assessed by Franklin’s functional constraint metrics.

**Table 6:** Segregation of variants among affected and unaffected siblings across different family structures.

Family Type	Affected Child—Variant Status	Unaffected Child—Variant Status
Two-child family (1 affected, 1 unaffected) *n* = 9	***ABCC8*:p.Ser1053Asn**	Absent
	***ABCC8*:p.Ala726Thr**	Absent
	Absent	***ANKRD11*** **:p.Arg840Gln**
	*HUWE1*:p.Asn483Ser	Absent
	*RAI1*:p.Gly90Ala	*RAI1*:p.Gly90Ala
	*SHANK2*:p.Arg818His	Absent
	*SHANK3*:p.Ile320Thr *	Absent
	Absent	***SLC9A9*:p.Asn43Lys**
	*STIL*:p.His985Arg	Absent
	Absent	*SYNE1*:p.Ser4596Thr
	Absent	*SYNE1*:p.Lys4121Arg
	*SYNE1*:p.Glu4060Asp	Absent
	Absent	*SYNE1*:p.Lys4121Arg
	Absent	*TSC2*:p.Gly1787Ser
	Absent	***VPS13B*:,p.Val3780Leu**
		*NIPBL*:p.Ala179Thr
		*NIPBL*: p.Asn968Ser
Three-child family (1 affected, 2 unaffected) *n* = 2	*ANK2*:p.Ile3285Thr	Absent
	*ANK2*:p.Val3634Asp	Absent
	***CUL3*:p.Gln343Arg**	***CUL3*:p.Gln343Arg**/Absent
	*HDAC4*:p.Val759Ile	*HDAC4*:p.Val759Ile/Absent
	*KCNJ11*:p.Leu270Val	*KCNJ11*:p.Leu270Val
	Absent	*NIPBL*:p.Ala179Thr
	Absent	*NIPBL*:p.Asn968Ser
	*NRXN1*:c.*98A>G	*NRXN1*:c. * 98A>G/Absent
	*POGZ*:p.His1363Gln	*POGZ*:p.His1363Gln/Absent
	*RELN*:p.Val2372Met	*RELN*:p.Val2372Met
	absent	*TSC1*:p.His732Tyr /Absent
	absent	***TSC2*:p.Arg1529Gln**
Two-child family (2 affected) *n* = 1	*MYT1L*:c.1104C>A,p.Pro368=	-

*—two cases, bold—Variant uncertain significance (VUS), —*n*—number of families.

## Data Availability

Sequencing data are available from the corresponding author on reasonable request.

## Abbreviations

The following abbreviations are used in this manuscript:

GWAS	Genome-wide association studies
NGS	next-generation sequencing
UTR	untranslated region
CNV	Copy number variation
